# Using panel data to examine pregnancy attitudes over time

**DOI:** 10.18063/ijps.2015.01.007

**Published:** 2015

**Authors:** Heini Väisänen, Rachel K. Jones

**Affiliations:** 1 Department of Social Policy, London School of Economics and Political Science, Houghton Street, London WC2A 2AE, United Kingdom; 2 Research Division, The Guttmacher Institute, 125 Maiden Lane 7^th^ floor, New York, N.Y. 10038, USA

**Keywords:** fertility intentions, pregnancy avoidance, pregnancy planning, panel data

## Abstract

There is a lack of research examining changes in women's fertility attitudes over relatively short periods of time. The aim of this study was to determine whether and how women's attempts to get pregnant and their desire to avoid pregnancy changed over six months' time as well as which characteristics and circumstances were associated with these changes. Using multinomial regression, we analyzed two panels of data from a sample of approximately 3,000 U.S. adult women gathered within six months apart. Only 4% of the women were trying to get pregnant at both time points, but six percent went from trying to not or vice versa. Two-thirds reported a strong desire to avoid pregnancy at both points, but 9% transitioned from strong to not strong and an additional 7% transitioned from not strong to strong. Women who transitioned to a more serious romantic relationship were at increased risk of transitioning to trying to become pregnant and, not surprisingly, to a weaker pregnancy avoidance. Some of the variables we tested, including changes in employment status and race/ethnicity, were associated with one outcome but not the other. The results highlight the importance of taking a holistic perspective of women's lives when studying pregnancy intentions and in reproductive health care services such as contraceptive counseling. Context matters and it may change rapidly.

## 1. Introduction

About half of the pregnancies in the United States are unintended (Finer and Zolna, 2014). This figure has remained relatively stable for two decades and has inspired researchers to study topics such as the determinants of fertility intentions (McQuilan, Greil, Shreffler *et al*., 2015; Reed and Mcbroom, 1995; Gatny, Kusunoki, and Barber, 2014), which groups of women are more likely to experience an unintended pregnancy (Finer and Zolna, 2014; Finer and Henshaw, 2006), to what extent individuals fulfill their desired family size (Berrington and Pattaro, 2014; Hartnett, 2014; Morgan and Rackin, 2010; Miller, Rodgers, and Pasta, 2010), and how well their intentions to have children within a specified time period are met (Miller, Rodgers, and Pasta, 2010; Heaton, Jacobson, and Holland, 1999; White and McQuilan, 2006). However, only a handful of studies have used longitudinal data to examine other fertility and family planning behaviors (Jones, Tapales, Lindberg *et al*, 2015). Instead, many cross-sectional studies assume that these attitudes are fairly constant over time. Even rarer are studies which use prospective longitudinal data to assess changes and continuity in short-term pregnancy attitudes over time.

Based on cross-sectional studies, we know that at any given point in time around five percent of U.S. women reported that they are trying to get pregnant (Jones, Mosher, and Daniels, 2012; McQuilan, Greil, and Shreffler, 2011). These women are often married, non-White, and are less likely to have children than women who are not trying to get pregnant (McQuilan, Greil, and Shreffler, 2011). Although many studies have noted that a dichotomous assessment of trying versus not trying does not describe the variety of pregnancy intentions (McQuilan, Greil, and Shreffler, 2011; Morgan, 1982; Santelli, Rochat, Hatfield-Timajchy *et al*, 2003), there is little research examining which groups of women are less interested in avoiding pregnancy, whether and how these attitudes change over time and for whom they do so. Interestingly, many women who are not actively trying to become pregnant are also not actively trying to avoid it. One study found that a fifth of women who were not trying to become pregnant reported that it was only a little or not at all important to avoid pregnancy(Frost, Singh, and Finer, 2007). Similarly, McQuillan's (McQuilan, Greil, and Shreffler, 2011) study found that 23 percent of women were “okay either way” when asked about becoming pregnant.

There are few longitudinal studies that have examined changes in fertility intentions over time. However, some studies have used the National Survey of Families and Households (NSFH) to analyze change and stability in the desire to have children over a relatively long time period of six-years. Among a subsample of 1,440 respondents who had no prior births and were in their first marriage or never married at baseline, the majority of women and men were stable in their fertility intentions, though almost one in five reported a change in intentions between the first and second waves (Heaton, Jacobson, and Holland, 1999). A second study, restricted to those who wanted to have (more) children at baseline, also found that the majority of individuals were consistent in their fertility intentions (including following through with their wave 1 intention to have children by wave 2), but they also found that 15 percent transitioned to no longer intending to have children, and six percent became unsure (White and McQuilan, 2006). However, neither of these studies addressed the changes in women's decisions to actively try to become pregnant or avoid pregnancy in the short term.

Short-term fertility attitudes have been studied longitudinally using The Relationship Dynamics and Social Life (RDSL) survey, which collected weekly data from 1,003 women aged 18–19 at baseline and residing in a single Michigan county in 2008 and 2009. The data show that that while nine in ten of these women were strongly motivated to avoid pregnancy at each point in time, only seven in ten did so consistently over the two and a half year study period (Moreau, Hall, Trussell, *et al*, 2013). These attitudes can influence contraceptive use: women who place little or no importance on avoiding pregnancy use less effective methods and use methods inconsistently (Frost, Singh, and Finer, 2007; Moreau, Hall, Trussell *et al*, 2013). This study is one of the few examining short-term changes in pregnancy attitudes, but is unfortunately limited to only young women and is not nationally representative.

Given the relatively small number of studies that have used longitudinal data to examine family planning behaviors, several gaps remain. The focus on young women in most studies likely reflects the recognition that early adulthood is a period of change across many domains. Particularly relevant when it comes to issues of family planning is that the majority of pregnancies to adolescent and young adult women are unintended, and this population is also less likely to use contraception consistently. Accordingly, understanding changes in fertility motivations and behaviors among this population is important. Yet researchers should not assume stability, or fail to examine changes in the family planning behaviors and attitudes of adult women. Childbearing is more normative among adult women, and patterns of fertility and pregnancy attitudes are likely to be distinctly different, and perhaps more variable, than those of adolescents and young adults. For example, one recent study using longitudinal data found that pregnancy avoidance attitudes were strongly associated with consistent contraceptive use and that this attitude changed for a majority of adult women over an 18-month time period (Jones, Tapales, Lindberg *et al*, 2015). Our study is the first to prospectively examine changes in fertility intentions and pregnancy avoidance attitudes, and the factors associated with these outcomes, among a national sample of adult U.S. women. While our study period is limited to the relatively short time period of six months, we find that these outcomes change for non-negotiable proportions of women.

Following the theory of conjunctural action (TCA) (Johnson-Hanks, Bachrach, Morgan *et al*, 2011), we expect women to have different attitudes towards pregnancies at different stages of their lives, as past fertility and other life events shape the behavior of individuals. Furthermore, the theory implies that women's reproductive behavior differs depending on their social class: women with low education more often start childbearing before marriage or stable employment than women with high education. Thus, examining whether change in short-term fertility goals and attitudes is associated with socio-demographic characteristics and changes in these circumstances, is of interest. We tested whether pregnancy attitudes changed in response to change in relationship or employment status, or whether background characteristics such as age, education, number and age of children, and race/ethnicity were associated with these attitudes.

## 2. Data and Methods

Data for this analysis come from Continuity and Change in Contraceptive Use (CCCU) Study, which was administered online to a national sample of women aged 18–39. To best capture women at risk of pregnancy, the sample only included women who ever had vaginal sex with a man, were not pregnant, and who herself or whose main male sexual partner had not been sterilized. In late 2012, 11,365 women were invited to participate; 6,658 (59 percent) answered the four screening items; 4,634 were eligible and completed the survey. A subsequent survey was conducted with the same women six months later, and 69 percent participated. Women who dropped out were younger (average age 28 rather than 29 among those who participated), less educated (32 percent had a college degree compared to 46 percent among those who stayed), less often White (57 percent vs. 66 percent) and childless (44 percent vs 52 percent). In this paper we study the 3,041 women who participated in both waves and were not pregnant at Wave 2.

We examine two outcome measures: whether women were trying to get pregnant and how much they wanted to avoid pregnancy. All women were asked, *“Which of the following best describes your current plans regarding having a(nother) baby?”* One of the response categories was “I am trying to get pregnant now.” Respondents were classified according to “never tried,” “constantly trying,” “stopped trying” (women who first reported trying, but were not trying at follow-up)and the opposite cases as “started trying.”

Respondents were also asked, *“How important is it to you to AVOID becoming pregnant now?”* and provided with a 6-point scale where 1 indicated “not at all important” and 6 “very important.” Women reporting values 4-6 at both waves were classified as having “consistently strong” pregnancy avoidance, women reporting values 1-3 as “never strong,” women reporting values 4-6 at first wave but 1-3 at second wave were classified as “became weaker,” and the opposite as “became stronger.” Preliminary analyses explored several coding schemes and resulted in largely the same findings.

Our explanatory variables included, firstly, changes in union status, which was classified as: no change; stronger union (for those who got married, started cohabiting or dating); and union dissolution (including divorce, dissolution of cohabiting union and transitioning from dating to single).

Employment status had three categories: not employed, employed part-time (<35 hours/week) and employed full-time (≥35 hours/week). Change in employment status was described using categories: “more work” (transitioning from no job to part- or full-time; or from part-time to full-time) and “less work” (transitioning from a part- or full-time job into unemployment; or from full-time to part-time).The survey did not assess whether women who were not employed had been laid off, were on leave, or were not working by choice.

Our analyses also include the baseline characteristics of age, race/ethnicity, education, parity, and the age of the youngest child in the household. Sample distributions of all variables are provided in Table 1. Values are reported at each wave for time variant and at baseline for time invariant ones.

We analyzed the data using descriptive statistics and multinomial regression. Firstly, independent variables were tabulated against the two outcome variables. Multinomial regression analyses with changes in trying (never trying as the base outcome); and pregnancy avoidance (consistently strong as the base outcome) as the outcome variables were conducted. The covariates that were not significant at 10% level were excluded from the final models. We also tested whether education interacted with any of the other characteristics, but the interactions were not statistically significant. The results were illustrated by calculating fitted probabilities using average marginal effects at representative values (Williams, 2012) for the outcome categories of interest; that is for having experienced a change in pregnancy avoidance or in trying to become pregnant. The probabilities were calculated for each explanatory variable by treating all respondents as though they had the characteristic of interest, say they experienced a union dissolution, leaving the values of all other variables as observed. The same calculation was subsequently conducted for each of the categories of the explanatory variable; that is also to “no change in relationship status” and “relationship became stronger”, for example. The average of these marginal effects became the probability of having experienced a change in pregnancy avoidance or in trying to become pregnant (Williams, 2012). We present the results as the predicted probabilities with 95 per cent confidence intervals.

## 3. Results

Four percent of women decided to start trying to get pregnant and two percent stopped trying without getting pregnant between baseline and follow-up studies (Table 2). Being in a romantic relationship that moved to “the next stage” was associated with starting to try to get pregnant more often (5 percent of women) than union dissolution (3 percent). Consistently working part-time was associated with starting (5 percent) and stopping trying (3 percent). Five to six percent of women who were aged 25 to 29 years, had high school education, had one child, had infants or toddlers, or were Black, started trying to get pregnant between the waves compared to two to four percent of women in the other categories of these covariates.

Nine percent of women transitioned from strong to not strong avoidance and seven percent from not strong to strong (Table 3). Women who got married or started dating or cohabiting transitioned to weaker pregnancy avoidance more often (12 percent) than women who experienced a union dissolution (7 percent). Although women working part-time were most likely to transition in either direction when it came to trying, the same group was the least likely to report a change in pregnancy avoidance. Women in their late 20s and early 30s experienced changes in pregnancy avoidance more often than others confirming that when it comes to pregnancy attitudes, adult women experience more change than adolescents. Women with high school diploma or less more often shifted to a weaker avoidance than other women (12–13 percent vs 8–10 percent). Parous women shifted more often to weaker avoidance than childless women (8 percent vs 11 percent), and mothers of infants were more likely to transition into either direction than women with older children. Race/ethnicity was not significantly associated with this outcome.

**Figure 1:**
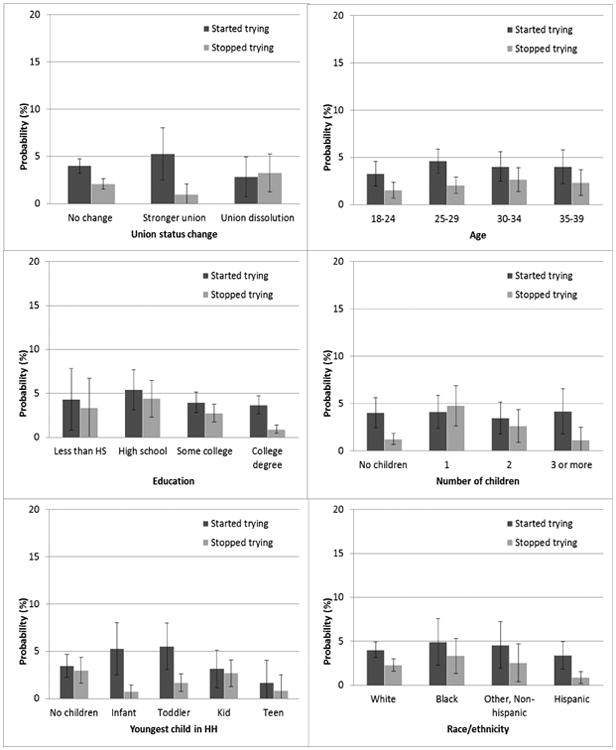
Changes in trying, fitted probabilities (%)* with 95% confidence intervals. * Calculated based on multinomial regression comparing outcomes never trying (reference), consistently trying, stopped trying, started trying. Tables including coefficients and p-values available on request.

**Figure 2:**
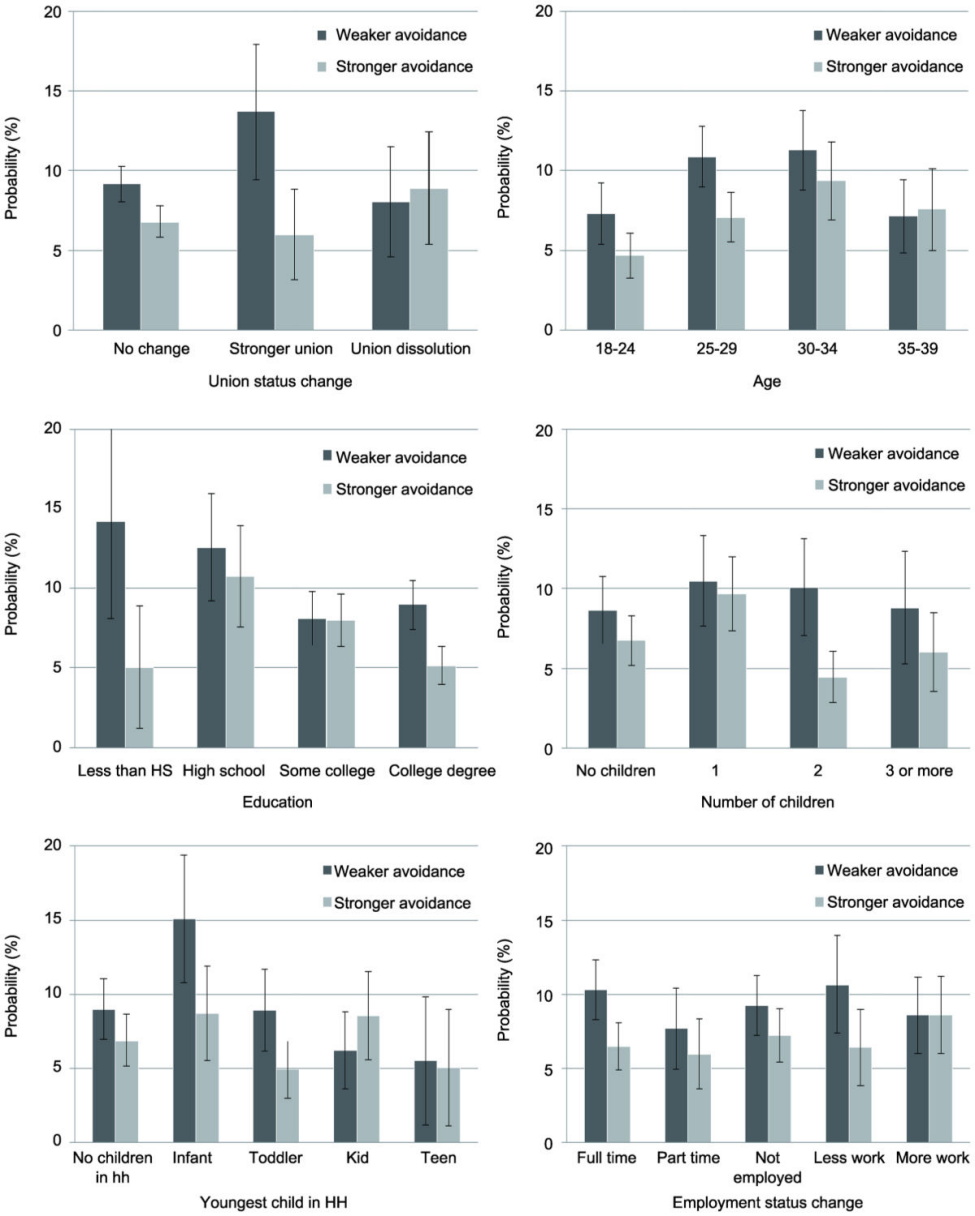
Changes in avoidance, fitted probabilities (%)* with 95% confidence intervals * Calculated based on multinomial regression comparing outcomes consistently strong (reference), never strong, became weaker and became stronger. Tables including coefficients and p-values available on request.

Findings using the multivariate analyses were similar to the bivariate analyses although fewer differences were statistically significant. Change in employment status was excluded from the model estimating the likelihood of experiencing changes in trying to get pregnant, and race/ethnicity from the pregnancy avoidance model. None of the interaction effects between education and the other covariates were significant at 10 percent level, and were thus not included. See Supplementary Online Materials A Tables A1 and A2 for full results of the models. Fitted probabilities, which were calculated based on the models, are shown below in Figures 1 and 2.

Figure 1 shows the fitted probabilities for changes in trying to get pregnant based on the multinomial model. Again, transitioning into a stronger union or not changing one's union status was associated with starting rather than stopping trying; women in their late 20s were more likely to start than stop trying; and mothers of infants and toddlers were relatively likely to start trying to get pregnant. Those who had a college degree more often started than stopped trying. Being childless or having at least three children was associated with a higher probability to start trying. Hispanic and White women were less likely to stop trying and more likely to start than other racial or ethnic groups.

Figure 2 shows the fitted probabilities of experiencing a change in pregnancy avoidance based on the multinomial model. The directions of associations were similar to the model where transitions in trying to get pregnant were studied for most variables. Moving to the “next stage” in one's union was associated with a relatively high (14 percent) probability in transitioning into weaker pregnancy avoidance. Women in their late 20s and early 30s had a higher probability of transitioning into weaker avoidance than other women. Women whose youngest child was an infant had a markedly higher probability of transitioning into weaker pregnancy avoidance compared to women with older children.

Unlike in the model measuring changes in trying, women who had less than a high school education had relatively high probability of transitioning into a weaker avoidance (14 percent), but women with a college degree were also more likely to transition into a weaker than into a stronger avoidance (9 percent vs 5 percent). Women with at least two children had a higher probability of transitioning into a weaker than stronger avoidance. Constantly working full-time women were more often associated with transitioning into a weaker avoidance than into a stronger one (Figure 2).

## 4. Discussion

While strong pregnancy avoidance and not trying to get pregnant was the norm for women in our sample, our results show that pregnancy attitudes change for a non-negotiable minority of women over a relatively short period of time. Perhaps not surprisingly, pregnancy avoidance showed more movement than efforts to get pregnant. Pregnancy avoidance has a behavioral element; as many women who have a strong desire to avoid pregnancy are likely to engage in practices to prevent this from happening, but it is less exclusive than those women who were reportedly actively trying to get pregnant.

Women in the lowest level of education were relatively likely to transition to weaker pregnancy avoidance, but less often into trying to get pregnant, whereas women with at least college degree had both higher likelihood of transitioning into weaker avoidance and starting to try. This may reflect different strategies of planning childbearing. According to TCA, after having decided to start a family, women with higher education are more likely to change their behavior beyond just not using contraception, for instance, by optimizing the timing of intercourse. Women from less advantaged backgrounds may take a more informal approach to childbearing, for example, accepting a pregnancy even when it is unplanned or alternatively, stopping contraception to show commitment to their partner (Johnson-Hanks, Bachrach, Morgan *et al*, 2011). Thus, there may be a higher likelihood of reporting trying to get pregnant among those with higher education compared to those with lower education, even while both groups' report a similarly weaker pregnancy avoidance attitude. Interestingly, no significant interactions between education and other covariates were found, although TCA suggests otherwise. It may have been partly due to the small sample size in our study.

Some of the associations were not surprising, such as transitioning into a stronger union being associated with weaker avoidance and trying to get pregnant. However, given that employment situation is often associated with fertility intentions in the literature (Becker, 1991; Chibber, Biggs, Roberts *et al*, 2014), it is interesting that changes in hours worked did not have a clear association with pregnancy attitudes. It may be that women interpret such changes as favorable or unfavorable depending on their other life circumstances. In addition, as the data did not assess whether women who reported working no hours in the week prior to the interview were on leave, not working by choice or had been laid off, it may be that in some cases we did not capture the kind of change that affects pregnancy planning with this variable.

Young women (aged 18 to 24) were less likely to transition in any direction in their pregnancy attitudes compared to older women. These patterns might reflect that younger women are more often pursuing education, stable employment and relationships thus, motivated to postpone childbearing confirming our hypothesis that there is more fluctuation in these attitudes among older women By contrast, women in their late 20s often transitioned into weaker pregnancy avoidance and started trying, which suggests that this is seen as a preferred age to have children.

Women who had young child(ren) more often reported shifting to weaker pregnancy avoidance and transitioning into trying to get pregnant. These women may wish to have their children relatively closely spaced.

These results highlight the importance of taking a holistic perspective of women's lives when studying pregnancy attitudes. Since we know that these attitudes are associated with consistency in contraceptive use (Frost, Singh, and Finer, 2007; Moreau, Hall, Trussell *et al*, 2013), this should be taken into account when contraceptive counseling is given. As pregnancy attitudes may change rapidly, women should know how to adjust their contraceptive use accordingly. This result also has a methodological implication: cross-sectional studies may not capture the entire story of pregnancy attitudes, as these studies assume that these measures are fairly stable over time.

There were limitations in this study. Women who were lost to attrition between waves were younger and less educated than women who stayed. However, if we observe this much change even among our sample of women probably leading more stable lives, there is no reason to expect that the associations would be weaker in a less biased sample. Moreover, we lacked information of partner's characteristics which may affect pregnancy intentions (Chibber, Biggs, Roberts *et al*, 2014). A larger sample size would have permitted a more detailed examination between different types of transitions in attitudes. Although the partner's characteristics such as his occupation or age may have influenced respondents' pregnancy attitudes, this information was not collected and in turn, we were unable to control for these characteristics. Similarly, it may be that the association between changes in relationship status and the outcome depend on whether the women are in their first or subsequent union and on the duration of the partnership. However, this information was not collected either. Future studies on the topic should consider measuring and studying these characteristics.

The strengths on the study include the innovative study design exploring rarely studied associations between changes in women's lives and fertility intentions. Moreover, there are very few existing longitudinal studies at the national level measuring adult women's fertility attitudes prospectively.

## Supplementary Material

Supplemental Files

## Figures and Tables

**Table 1:** Pregnancy attitudes and socio-demographic characteristics at baseline (and Wave 2 for time varying covariates), %

	Baseline	Wave 2	Both waves	N (baseline)
PREGNANCY ATTITUDES				
Trying	6	8	4	192
Not trying	94	92	90	2827
Total	100	100	94	3019
Weak avoidance	23	26	16	697
Strong avoidance	77	74	68	2327
Total	100	100	84	3024

UNION STATUS				
Married	45	46	44	1,378
Cohabiting	20	20	16	621
Dating	21	20	14	653
Single	13	14	9	389
Total	100	100	82	3,041

EMPLOYMENT				
Not employed	35	34	28	1,030
Less than full-time	24	21	13	729
Full time	41	45	35	1,218
Total	100	100	76	2977

AGE				
18–24	27			807
25–29	34			1,036
30–34	21			640
35–39	18			558
Total	100			3,041

PARITY				
0	53			1,609
1	20			592
2	18			532
3 or more	10			299
Total	100			3,032

YOUNGEST CHILD IN HOUSEHOLD				
No children in hh	51			1,562
Infant (0–12 months)	14			430
Toddler (1–3 yrs)	19			574
Kid (4–12 yrs)	12			355
Teen (13–19 yrs)	4			120
Total	100			3,041

RACE/ETHNICITY				
White	65			1,978
Black	9			273
Hispanic	8			254
Other	18			536
Total	100			3,041
EDUCATION				
Less than high school	5			145
High school	14			412
Some college	36			1,105
BA or higher	45			1,379
Total	100			3,041

**Table 2:** The bivariate associations between the explanatory variables and trying (%)

Trying to become pregnant	Never trying	Consistently trying	Started trying	Stopped trying	Total	N
TOTAL	90	4	4	2	100	3,000

UNION STATUS					*p* = 0.001	
No change	89	5	4	2	100	2,466
Stronger union	93	1	5	1	100	280
Union dissolution	91	2	3	4	100	254

EMPLOYMENT					*p* = 0.044	
Full time	91	4	3	2	100	348
Part time	87	5	5	3	100	775
Not working	94	2	2	2	100	380
Less work	89	5	4	2	100	1,005
More work	91	4	4	1	100	481

AGE AT BASELINE					*p* = 0.010	
18–24	93	2	3	2	100	802
25–29	89	4	5	2	100	1,021
30–34	87	6	4	3	100	630
35–39	89	5	4	2	100	547

EDUCATION AT BASELINE					*p* = 0.004	
Less than high school	87	6	4	3	100	143
High school	85	5	5	4	100	401
Some college	90	4	4	3	100	1,090
College degree	91	4	4	1	100	1,366

PARITY (Wave II)					*p* = 0.001	
0	90	5	3	1	100	1,593
1	85	6	5	4	100	581
2	91	2	4	2	100	526
3 or more	92	2	5	1	100	300

YOUNGEST CHILD IN HH					*p* = 0.001	
No children in hh	90	5	4	2	100	1,540
Infant (0–12 months)	92	2	5	1	100	425
Toddler (1–3 yrs)	86	6	6	2	100	569
Kid (4–12 yrs)	89	3	3	4	100	348
Teen (13–19 yrs)	94	3	2	1	100	118

RACE/ETHNICITY					*p* = 0.029	
White	90	4	4	2	100	1,953
Black	88	3	5	4	100	267
Other, Non-Hispanic	91	3	4	2	100	252
Hispanic	89	7	4	1	100	528

**Table 3:** The bivariate associations between the explanatory variables and pregnancy avoidance (%)

Pregnancy avoidance	Consistently strong	Never strong	Became weaker	Became strong	Total	N
TOTAL	68	16	9	7	100	3,011

UNION STATUS					*p* < 0.001	
No change	66	17	9	7	100	2,477
Stronger union	73	9	12	6	100	279
Union dissolution	73	10	7	9	100	255

EMPLOYMENT					*p* < 0.001	
Full time	65	19	10	6	100	1,006
Part time	76	11	7	6	100	387
Not working	64	18	11	8	100	780
Less work	68	15	10	7	100	349
More work	72	12	8	8	100	478

AGE AT BASELINE					*p* < 0.001	
18–24	79	8	7	5	100	801
25–29	65	17	11	7	100	1,023
30–34	59	20	12	9	100	635
35–39	65	21	7	7	100	552

EDUCATION AT BASELINE					*p* < 0.001	
Less than high school	59	22	13	5	100	143
High school	58	19	12	11	100	405
Some college	69	15	8	8	100	1,097
College degree	70	15	10	5	100	1,366

PARITY (Wave II)					*p* < 0.001	
0	71	15	8	6	100	1,593
1	58	20	11	11	100	582
2	69	15	11	5	100	529
3 or more	67	16	11	7	100	303

YOUNGEST CHILD IN HH					*p* < 0.001	
No children in hh	70	16	8	6	100	1,542
Infant (0–12 months)	60	15	16	9	100	428
Toddler (1–3 yrs)	65	20	10	6	100	570
Kid (4–12 yrs)	68	15	7	10	100	353
Teen (13–19 yrs)	79	11	5	5	100	118

RACE/ETHNICITY					*p* = 0.759	
White	67	17	9	7	100	1,958
Black	65	16	12	7	100	270
Other, Non-Hispanic	72	14	8	6	100	249
Hispanic	67	15	9	8	100	534
