# Supplementary material for: Using panel data to examine pregnancy attitudes over time
Source: Int J Popul Stud. Author manuscript; Available in PMC 2016 Aug 23. (PMC4994192; doi:10.18063/ijps.2015.01.007)
Supplement: Supplemental Files [file NIHMS796652-supplement-Supplemental_Files.pdf]

International Journal of Population Studies

Supplementary Online Material A: Results of the Multinomial Logistic Regression Models

Table A1. Changes in trying to become pregnant, results of the multinomial logit model as odds ratios with 95% confidence intervals.

Outcome (reference category: never trying)		Consistently trying		Started trying		Stopped trying	
Variable	*Category*	OR	CI 95%	OR	CI 95%	OR	CI 95%
Relation-ship status	*No change*	1.00		1.00		1.00	
	Stronger union	0.13**	(0.03,0.55)	1.26	(0.69,2.27)	0.44	(0.13,1.48)
	Union dissolution	0.34*	(0.14,0.85)	0.68	(0.31,1.51)	1.52	(0.73,3.16)
Age at baseline	*18–24*	1.00		1.00		1.00	
	25–29	2.24**	(1.24,4.03)	1.49	(0.89,2.47)	1.42	(0.69,2.91)
	30–34	4.26***	(2.30,7.86)	1.35	(0.74,2.47)	1.90	(0.86,4.19)
	35–39	3.92***	(2.04,7.52)	1.32	(0.68,2.55)	1.65	(0.68,3.97)
Race or ethnicity	*White*	1.00		1.00		1.00	
	Black	0.74	(0.33,1.64)	1.24	(0.67,2.29)	1.47	(0.71,3.05)
	Other non-Hispanic	0.69	(0.31,1.53)	1.13	(0.59,2.17)	1.10	(0.42,2.86)
	Hispanic	1.76*	(1.12,2.77)	0.85	(0.50,1.46)	0.37*	(0.15,0.91)
Parity	*No children*	1.00		1.00		1.00	
	One child	0.58	(0.30,1.14)	1.02	(0.50,2.07)	3.96***	(1.83,8.60)
	Two children	0.15***	(0.06,0.34)	0.79	(0.36,1.73)	2.00	(0.78,5.15)
	Three or more	0.076***	(0.02,0.24)	0.93	(0.39,2.22)	0.83	(0.21,3.35)
Youngest child in house-hold (hh)	*No children in hh*	1.00		1.00		1.00	
	Infant (0–12 months)	0.97	(0.38,2.47)	1.51	(0.70,3.28)	0.24*	(0.07,0.78)
	Toddler (1–3 yrs)	2.74**	(1.37,5.48)	1.71	(0.83,3.52)	0.60	(0.26,1.38)
	Kid (4–12 yrs)	1.06	(0.49,2.29)	0.91	(0.41,2.04)	0.89	(0.40,1.97)
	Teen (13–19 yrs)	0.93	(0.31,2.72)	0.47	(0.11,1.99)	0.26	(0.03,2.02)
Educa-tion at baseline	*Less than high school*	1.00		1.00		1.00	
	High school	0.77	(0.31,1.89)	1.26	(0.49,3.27)	1.32	(0.41,4.22)
	Some college	0.56	(0.24,1.33)	0.88	(0.35,2.19)	0.77	(0.25,2.36)
	College degree	0.42*	(0.18,1.00)	0.78	(0.31,1.99)	0.24*	(0.07,0.82)

Notes:
Total N=3000; Pseudo R-squared=0.067; Exponentiated coefficients; 95% confidence intervals in parenthesis;
* p<0.05, ** p<0.01, *** p<0.001 describing statistical significance in relation to the reference category.

Table A2. Changes in pregnancy avoidance, results of the multinomial logit model as odds ratios with 95% confidence intervals.

Outcome (reference category: always strong)		Never strong		Became weaker		Became strong	
Variable	*Category*	OR	CI 95%	OR	CI 95%	OR	CI 95%
Relation-ship status	*No change*	1.00		1.00		1.00	
	Stronger union	0.53**	(0.34,0.82)	1.41	(0.94,2.11)	0.82	(0.48,1.42)
	Union dissolution	0.51**	(0.33,0.79)	0.79	(0.48,1.30)	1.18	(0.73,1.90)
Employ-ment	*Full-time job*	1.00		1.00		1.00	
	Part-time job	0.54**	(0.37,0.78)	0.64	(0.40,1.00)	0.78	(0.47,1.30)
	Not working	0.94	(0.71,1.25)	0.88	(0.62,1.26)	1.09	(0.73,1.65)
	Less work	0.85	(0.60,1.22)	1.00	(0.66,1.54)	0.96	(0.57,1.61)
	More work	0.67*	(0.48,0.93)	0.77	(0.51,1.16)	1.23	(0.79,1.91)
Age at baseline	*18–24*	1.00		1.00		1.00	
	25–29	2.78***	(2.01,3.84)	1.87***	(1.31,2.67)	1.92**	(1.27,2.91)
	30–34	4.10***	(2.87,5.85)	2.22***	(1.47,3.34)	2.90***	(1.82,4.62)
	35–39	4.43***	(3.05,6.43)	1.32	(0.81,2.14)	2.21**	(1.31,3.73)
Parity	*No children*	1.00		1.00		1.00	
	One child	1.00	(0.68,1.47)	1.29	(0.80,2.10)	1.53	(0.92,2.52)
	Two children	0.45***	(0.29,0.70)	0.99	(0.58,1.69)	0.56	(0.30,1.03)
	Three or more	0.40***	(0.24,0.67)	0.85	(0.46,1.59)	0.74	(0.37,1.48)
Youngest child in house-hold	*No children in hh*	1.00		1.00		1.00	
	Infant (0–12 months)	1.42	(0.91,2.23)	2.02**	(1.21,3.38)	1.53	(0.86,2.73)
	Toddler (1–3 yrs)	1.36	(0.91,2.03)	1.04	(0.62,1.73)	0.74	(0.41,1.33)
	Kid (4–12 yrs)	0.84	(0.54,1.28)	0.66	(0.37,1.17)	1.18	(0.69,2.02)
	Teen (13–19 yrs)	0.63	(0.33,1.19)	0.53	(0.22,1.26)	0.63	(0.26,1.52)
Educa-tion at baseline	*Less than high school*	1.00		1.00		1.00	
	High school	0.80	(0.48,1.32)	0.87	(0.48,1.59)	2.11	(0.90,4.96)
	Some college	0.52**	(0.33,0.84)	0.45**	(0.26,0.80)	1.27	(0.56,2.91)
	College degree	0.34***	(0.21,0.55)	0.45**	(0.25,0.81)	0.73	(0.31,1.71)

Notes:

Total N=2996; Pseudo R-squared=0.05; Exponentiated coefficients; 95% confidence intervals in parenthesis; * p<0.05, ** p<0.01, *** p<0.001, describing statistical significance in relation to the reference category.
